# Regulation of Drosophila Lifespan by *bellwether* Promoter Alleles

**DOI:** 10.1038/s41598-017-04530-x

**Published:** 2017-06-23

**Authors:** Júlia Frankenberg Garcia, Mary Anna Carbone, Trudy F. C. Mackay, Robert R. H. Anholt

**Affiliations:** 1 Program in Genetics, W. M. Keck Center for Behavioral Biology, Department of Biological Sciences, North Carolina State University, Raleigh, North Carolina USA; 2 School of Biosciences and Medicine, Faculty of Health and Medical Sciences, University of Surrey, Guildford, UK

## Abstract

Longevity varies among individuals, but how natural genetic variation contributes to variation in lifespan is poorly understood. *Drosophila melanogaster* presents an advantageous model system to explore the genetic underpinnings of longevity, since its generation time is brief and both the genetic background and rearing environment can be precisely controlled. The *bellwether* (*blw*) gene encodes the α subunit of mitochondrial ATP synthase. Since metabolic rate may influence lifespan, we investigated whether alternative haplotypes in the *blw* promoter affect lifespan when expressed in a co-isogenic background. We amplified 521 bp upstream promoter sequences containing alternative haplotypes and assessed promoter activity both *in vitro* and *in vivo* using a luciferase reporter system. The AG haplotype showed significantly greater expression of luciferase than the GT haplotype. We then overexpressed a *blw* cDNA construct driven by either the AG or GT haplotype promoter in transgenic flies and showed that the AG haplotype also results in greater *blw* cDNA expression and a significant decrease in lifespan relative to the GT promoter haplotype, in male flies only. Thus, our results show that naturally occurring regulatory variants of *blw* affect lifespan in a sex-specific manner.

## Introduction

Lifespan is highly variable among individuals and is determined by the complex interplay between genetic and environmental factors^1, 2^. Evolutionary theories regarding genetic limitations on lifespan have proposed the persistence of deleterious alleles in the genome that are activated at later age after reproduction^3–6^, or antagonistic pleiotropy of alleles that are beneficial early in life and deleterious later on^7, 8^. Oxidative stress^9–11^, genomic instability^12–15^, telomere length^16–18^ and DNA repair mechanisms^19–22^ have been implicated as mechanisms that affect aging and longevity. However, little is known about the mechanisms by which naturally occurring allelic variants within a population affect variation in lifespan.

Oxidative stress occurs through the production of reactive oxygen species (ROS) as a byproduct of mitochondrial oxidative phosphorylation^23, 24^. Previously, single nucleotide polymorphisms (SNPs) in the promoter region of the Drosophila *bellwether* (*blw*) gene have been associated with differences in lifespan between control flies and long-lived lines of flies originally selected for delayed reproduction^25, 26^. This study showed that all four of the long-lived lines selected for postponed reproduction that were genotyped for the *blw* promoter were fixed for the GT haplotype, but this haplotype was lost or at very low frequency in the five controls that were genotyped for the *blw* promoter^25^. The *blw* gene encodes the α subunit of mitochondrial ATP synthase, suggesting that sequence variants in this gene could give rise to subtle differences in metabolic rate which could affect the production of ROS during the organism’s lifespan^27^. Here, we show that alternative haplotypes in the promoter region of *blw* result in different levels of gene expression and that introduction of a transgenic *blw* construct driven by these alternative promoters in a co-isogenic background causes a profound sex-specific effect on lifespan. These results provide a mechanistic link between lifespan and allelic variation in a central metabolic gene.

## Results

### RNAi-mediated inhibition of *blw* expression results in lethality

**Figure 1:**
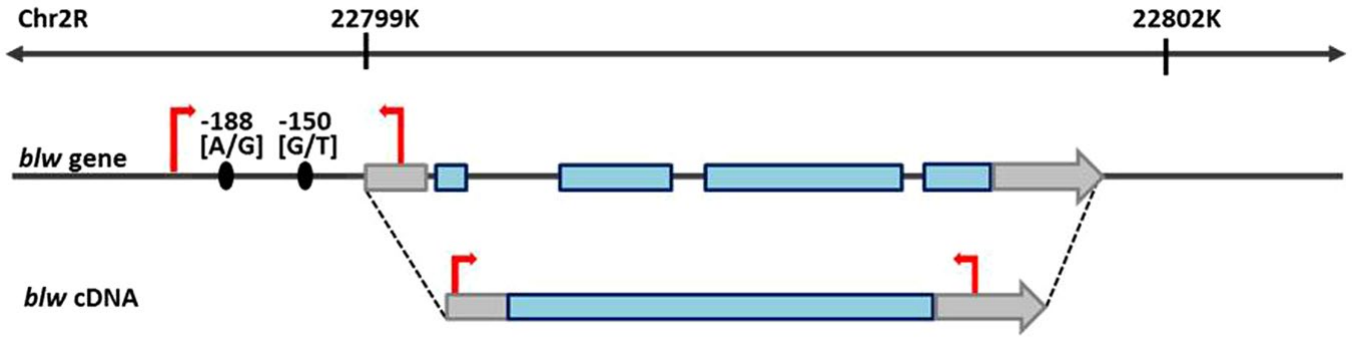
Diagram of the structure of the *D. melanogaster blw* gene. The *blw* gene is located on Chr*2R*:22,799,099…22,802,180 [+] and generates a single transcript composed of a 5′-UTR (gray box), four exons (blue boxes) and a 3′-UTR (gray arrow) (Flybase.org). The two SNPs are located 150 bp [G/T] and 188 bp [A/G] upstream of the *blw* transcriptional start site (gray box). The location of PCR primers used to generate the *blw*-promoter or *blw*-cDNA are indicated by red arrows.

The Drosophila *blw* gene is located on chromosome *2* (Chr2R:22,799,099…22,802,180 [+]) and generates a single transcript composed of a 5′-UTR (105 bp), 4 exons (66 bp, 581 bp, 802 bp and 210 bp) and a 3′-UTR (488 bp) (Fig. 1)^28^. Prior to examining regulation of *blw* expression under alternative promoters, we assessed the effect of RNAi-mediated knockdown of *blw* expression by crossing flies homozygous for a *UAS-blw-RNAi* transgene to flies that drive *GAL4* expression under ubiquitin or actin promoters. No embryos developed when *blw* was knocked down with the actin promoter. When *blw* was knocked-down using the ubiquitin promoter, flies reached the pupal stage but did not eclose from the pupal cases. These results demonstrate that *blw* is an essential gene for development and viability of *D. melanogaster*.

### Functional analysis of the *blw* promoter region

**Figure 2:**
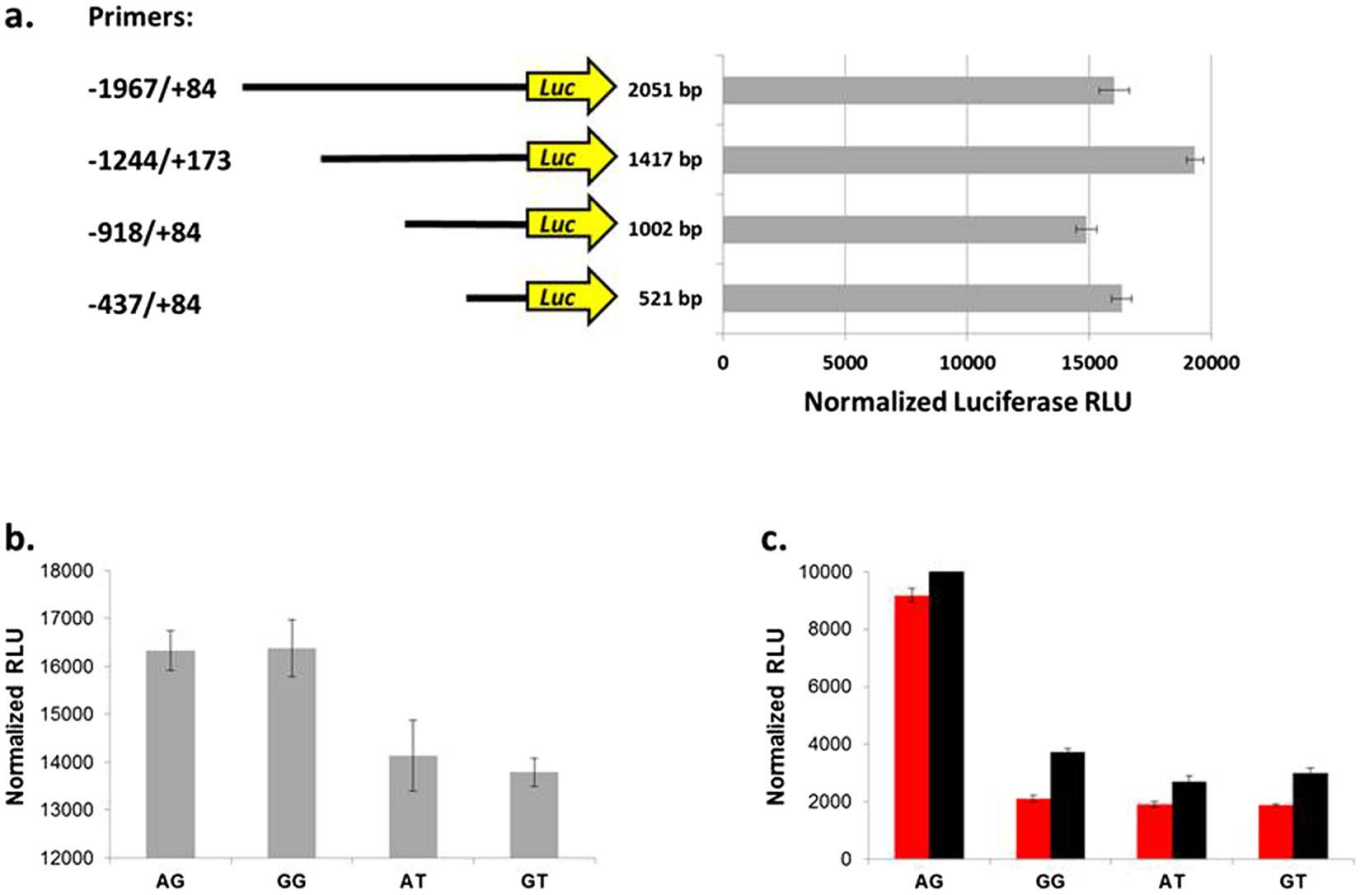
Relative strength of the putative promoter region of the *blw* gene. (**a**) Four different lengths of the *blw*-promoter region harboring the AG haplotype were cloned in the pGL3-basic vector and expressed in Drosophila S2 cells. Primer locations are indicated with respect to the transcriptional start site of the *blw* gene (see Fig. 1). Black lines represent the promoter length and the yellow arrow represents the luciferase reporter gene. (**b**) The 521 bp *blw*-promoter region harboring the AG, GG, AT and GT haplotypes were cloned in the pGL3-basic vector and expressed in Drosophila S2 cells. (**c**) The same promoters were expressed in flies using the *Gal4-UAS* binary expression system. For each experiment, protein lysates were extracted and luciferase expression was measured and normalized (relative light units; RLU). Gray bars represent the Drosophila S2 cells (Panels a and b). Female flies are represented by red bars and males by black bars (Panel c). Error bars are standard errors of the mean.

To examine whether alternative haplotypes in the *blw* promoter affect gene expression, we generated promoter constructs of different lengths containing the AG haplotype and tested their activity in an *in vitro* firefly luciferase reporter system. All *blw* promoter constructs encompass the 56 bp region containing the GAGA and Adf-1 elements essential for promoter activity^29^. All constructs were effective in driving luciferase expression (Fig. 2a) and therefore we selected the shortest, 521 bp promoter region for further studies.

We cloned the four haplotypes of the 521 bp *blw* promoter region, −188A/−150G, −188A/−150T, −188G/−150G and −188G/−150T (designated AG, AT, GG and GT respectively), into the pGL3-basic-Luciferase vector and transfected Drosophila S2 cells. The AG and GG haplotypes showed substantially greater luciferase expression than the AT and GT haplotypes (*P* < 0.0001) (Fig. 2b).

Next, we asked whether the same difference would be replicated *in vivo*. We cloned the same promoters in a *pattB-Gal4-hsp70* vector and drove luciferase expression in transgenic flies by crossing flies homozygous for the *Gal4*-*blw*-promoter with flies carrying a *UAS*-luciferase construct. We measured luciferase activity in protein lysates from the F1, sexes separately, to compare the strength of each promoter (Fig. 2c). Here, the AG haplotype showed approximately 3-fold greater luciferase expression than the other haplotypes in both sexes (*P* < 0.0001).

We compared our *in vitro* and *in vivo* observations. Analyses of variance revealed a significant difference among the haplotypes and in promoter strength between the −150G and −150T SNPs both in cell culture and in flies (*P* = 0.0008 (*in vitro*); *P* < 0.001 (*in vivo*) Fig. 2). In both cases, the −150G allele results in stronger promoter expression. In contrast, the −188[A/G] appears to have no effect when expressed in cell culture (*P* = 0.823). When expressed in flies, however, the −188A allele is significantly stronger than the −188G allele (*P* < 0.0001). These results reveal the effect of each individual SNP on the strength of the *blw* promoter. Based on our *in vivo* results, we focused further experiments on the effects of the AG and GT haplotypes on lifespan.

### Overexpression of *blw* from the AG promoter shortens lifespan

**Figure 3:**
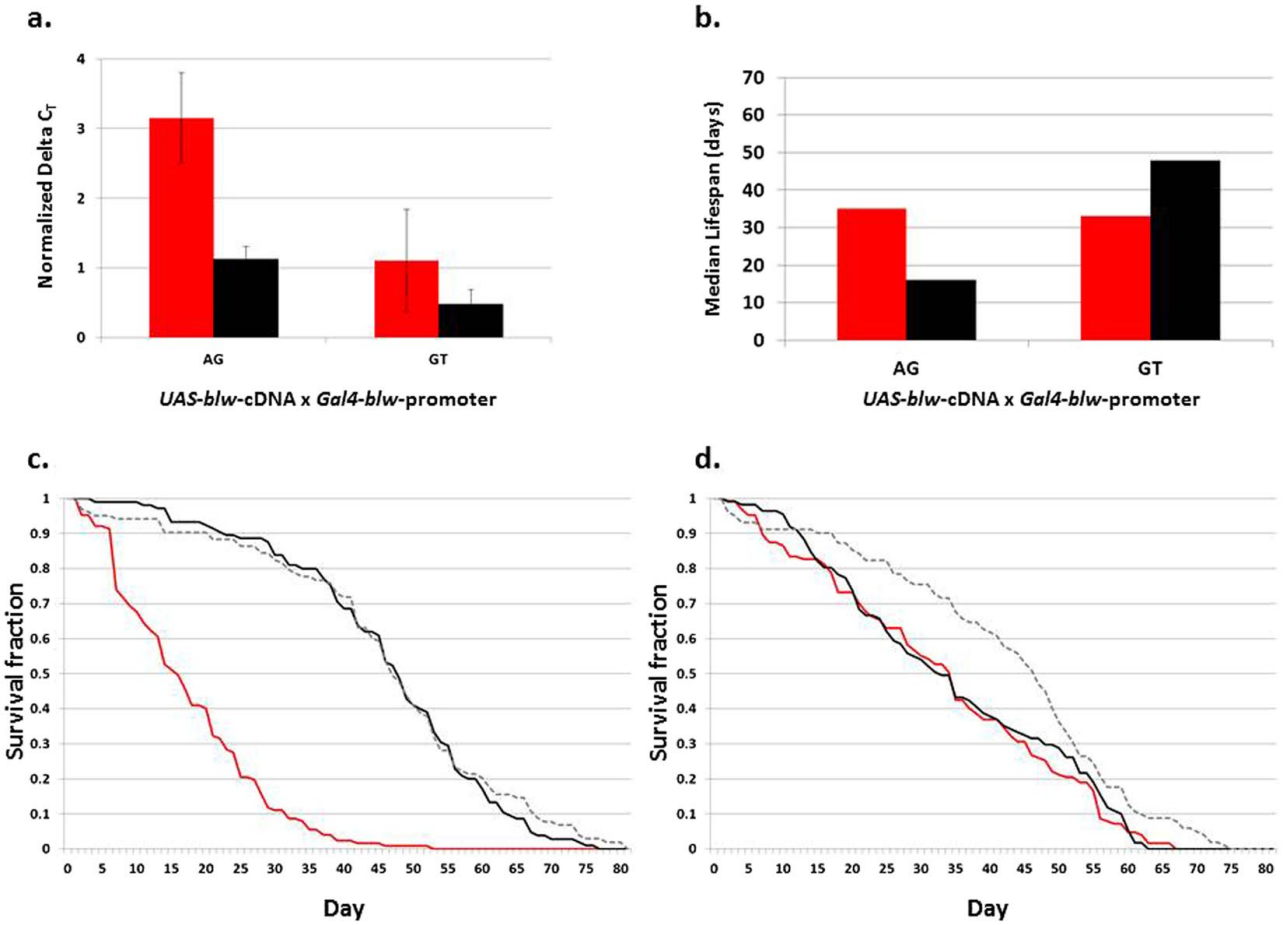
Effect of *blw*-cDNA overexpression on lifespan. (**a**) Normalized expression levels measured by quantitative RT-PCR of *blw*-cDNA when driven by the *blw*-AG and *blw*-GT promoters (red, females; black, males). (**b**) Median lifespan of flies that overexpress *blw*-cDNA from the *blw*-AG and *blw*-GT promoters (red, females; black, males). (**c**) Survival curves of male flies overexpressing *blw*-cDNA when driven by either the *blw*-AG (red) or *blw*-GT promoter (black). (**d**) Survival curves of female flies overexpressing *blw*-cDNA when driven by either the *blw*-AG (red) or *blw*-GT promoter (black). The control flies expressing the endogenous *blw* gene are shown by gray dotted lines.

We used the *Gal4-UAS* binary expression system^30, 31^ to investigate the effects of overexpression of a *blw* cDNA construct driven by promoters with either the AG or GT haplotype in a co-isogenic background. Quantitative real-time PCR showed that the promoter with the AG haplotype drives stronger *blw* cDNA expression than the GT haplotype (*P* = 0.01), in line with observations from our *in vivo* and *in vitro* luciferase reporter gene experiments (Fig. 3a). Flies in which *blw* cDNA expression is driven by the AG promoter, however, have a reduced median lifespan compared to flies in which *blw* cDNA expression is driven by the GT promoter (Fig. 3b). We fitted a mixed effects Cox model including the sex by haplotype term to the lifespan data. This analysis revealed a strong sex by haplotype effect (*P* < 6 × 10^−5^, Fig. 3b). Therefore we performed survival analyses for sexes separately. For each sex, we fitted another mixed effects Cox model, testing for significance of the differences of hazard between the two haplotypes and with controls. In males there was no difference between haplotype GT and control (HR = 0.99, *P* = 0.96), but the hazard for the GT haplotype was markedly higher than the AG haplotype (Fig. 3c, HR = 12.83, *P* = 2.47 × 10^−32^). By contrast, in females there was a significant effect between the AG and GT haplotypes with the controls (hazard ratio (HR) = 1.70 and *P* = 0.003 for AG versus control and HR = 1.69 and *P* = 0.004 for GT versus control) but no difference between the AG and GT haplotypes (HR = 0.99, *P* = 0.96; Fig. 3d).

## Discussion

Sex differences in lifespan have been observed in model organisms including *C. elegans, D. melanogaster*, and *Mus musculus* ^32, 33^. Sexual dimorphism in genetic architecture^34^ is a common feature of quantitative traits and has been documented for morphological^35–37^, behavioral^38, 39^, physiological^40, 41^, and life history traits^42, 43^, including lifespan^44, 45^, in Drosophila. The genetic architecture of Drosophila lifespan in particular, is sexually dimorphic^25, 45–50^. Here, we investigated the effects of allelic variants in the promoter region of *blw* on lifespan and showed that overexpression of the AG haplotype shortens male lifespan compared to its GT counterpart. Our luciferase-reporter assays revealed a significant difference in promoter expression between the AG and GT haplotypes both in cell culture and in flies.

The *blw* gene encodes the ATP synthase α subunit, essential for oxidative phosphorylation^27^, and it is therefore not surprising that inhibition of *blw* expression by RNAi results in lethality. This observation is in line with previous studies on *blw* mutant alleles, which showed larval growth defects affecting all tissues, DNA endoreplication defects, and larval lethality with no homozygous animals reaching the pupal stage^51^. A previous study showed that a *blw* mutant, generated by insertion of a transposable element in the 5′ UTR (*blw* ^KG05893^), reduced adipose tissue growth and triglyceride storage and increased ROS in third instar larval fat bodies^52^.

All four long-lived *D. melanogaster* lines (O-lines) selected for postponed reproduction that were genotyped for the *blw* promoter contain the GT haplotype, whereas this haplotype was lost or present at low frequency in all of the corresponding control base lines (B-lines)^25^. Assessment of feeding behavior, measured by a capillary feeding (CAFÉ) assay, showed that the B-lines consume more sucrose compared to the O-lines and that food consumption declines with age. The increased feeding behavior of the B-lines may correlate with increased metabolic rate and, therefore, shorter lifespan compared to the O-lines^25^. This corresponds with our results, since we observe a stronger promoter activity of the *blw*-*AG* haplotype (representative of the B-lines) resulting in higher expression levels of *blw*-cDNA, and a decreased male lifespan of the *UAS-blw-cDNA x Gal4-blw-AG* promoter lines.

Mechanisms of aging may involve metabolic regulation through the insulin signaling pathway, as evident from the effects of mutations in components of this pathway, including *foxo*, *InR* and *chico* ^53–56^. In addition, the major nutrient-signaling pathways, that depend on *mTOR* ^57–59^, *Sir2* ^60, 61^, and *insulin-like* ^62, 63^ genes, have been associated with extension of lifespan in flies subjected to dietary restriction. However, the benefits of dietary restriction on lifespan extension are eliminated by exposure to oligomycin, a specific inhibitor of mitochondrial ATP synthase^64^, implicating the electron transport chain.

Invadolysin, a lipid-droplet associated protein, interacts physically with three mitochondrial ATP synthase subunits: α (*bellwether*), β and δ^52^. Multiple proteomic screens have demonstrated that the ATP synthase subunits also interact with lipid droplets in Drosophila embryos, third instar larvae, and in human adipocytes^65^. Both *invadolysin* and *blw* mutants have defects in mitochondrial electron transport chain activity and thus produce high levels of ROS^52^. Furthermore, *invadolysin* mutants exhibit increased autophagy and decreased glycogen storage^66^. Together, these data suggest that *blw* plays a role in lifespan determination via its physical interaction with *invadolysin*.

Previous studies on aging in Drosophila have led to the discovery of additional genes that extend lifespan, including *mth* ^67–69^, *Indy* ^70, 71^, *InR* ^55, 72, 73^, *chico* ^56, 74, 75^, and *SOD* ^76–79^. Also, *bride of sevenless* (*boss*) null mutants have shortened lifespans, diminished locomotor performance and elevated ROS production^80^. In addition, *boss* mutant flies express higher levels of *blw* compared to control flies, further implicating a connection between decreased lifespan and increased metabolic rate, correlated with expression of *blw*.

It should be noted that our transgenic flies that overexpress *blw* from a cDNA construct still contain endogenous *blw*. The presence of the endogenous gene might amplify the deleterious effect of overexpression of *blw* under the AG promoter in males as it may allow overexpression of the transgene to surpass a critical threshold, which might not be reached in the absence of the endogenous gene.

It is tempting to speculate that greater expression of the *blw* ATP synthase α-subunit under the AG promoter may result in enhanced metabolic rate, generating more ROS, which results in shorter lifespan. In this scenario, the female sex environment would appear to be protective against the effects of the AG haplotype and metabolically generated oxidative stress. Although further experiments are necessary to consolidate or refute this hypothesis, our study demonstrates a link between allelic variation in the promoter of the *blw* gene and Drosophila lifespan.

## Methods

### *In-vitro* promoter luciferase assays

We used PCR to amplify four different lengths (521 bp, 1002 bp, 1417 bp and 2051 bp) of the promoter region, containing the AG haplotype, upstream of the *blw* coding region from genomic DNA using directional primers based on the Drosophila reference strain (line 2057) and cloned the amplicons into the *Kpn1/Xho1* multiple cloning site of the pGL3-basic vector (Promega). We screened colonies by *Kpn1/Xho1* double-digestion and Sanger sequencing to identify positive clones, and used site-directed mutagenesis to generate the other haplotypes using *Pfu* phusion HotStart Flex DNA polymerase (New England Biolabs). Following PCR-amplification the parental template was digested with *Dpn1*, and the DNA was transformed into JM109 competent cells (Promega). Clones were purified using the Qiagen MiniPrep kit (Qiagen) and validated by Sanger sequencing^81^.

*Drosophila* S2 cells were cultured at room temperature in Schneider’s *Drosophila* medium (Invitrogen) supplemented with 10% heat-inactivated fetal bovine serum (Invitrogen) and 100 μg/ml of gentamicin (Gibco). Cells were counted 24 h prior to transfection using a Countess Automated Cell Counter (Invitrogen) and 1 million cells were transferred to the wells of a 6-well plate. Each *blw* promoter construct was co-transfected with a *Renilla* luciferase vector as an internal transfection control (pGL4.74[hRluc/TK] vector; Promega, E6921) using Cellfectin II reagent (Invitrogen). Transfections were performed in triplicate. After incubation for 72 h at 28 °C protein lysates were extracted and subjected to a Dual-Glo luciferase assay (Promega). Firefly and *Renilla* luciferase activity were measured with a GloMax luminometer. The firefly luciferase activity was normalized against the *Renilla* luciferase activity for each sample and data were analyzed using SAS software version 9.3^82^. We performed an analysis of variance (ANOVA) for luciferase activity: *Y* = *μ* + *H* + *ε*, where Y is the observed value, μ is the mean, *H* is the promoter haplotype, and ε is the residual (error) variance. The normalized relative light units emitted by the assay revealed the strength of each promoter.

### *In-vivo* promoter luciferase assays

We excised the 521 bp *blw*-promoter inserts from the pGL3-basic vector with *Kpn1* and *BglII* and ligated them into the *pattB-Gal4-synaptobrevin-hsp70* vector (Addgene; Plasmid #46107) after excision of the *synaptobrevin* promoter with *BamH1* and *EcoR1*. Since the inserts and plasmids contained incompatible ends, cloning was achieved using the In-Fusion® HD Cloning Plus CE kit (Clontech) with the following primers to amplify the *blw*-promoter inserts: *blw*-InFusion-F, 5′-TTATGCTAGCGGATCTGGCGGCGTCCACATATA and *blw*-InFusion-R, 5′- CTTCATGTTGGAATTACTGTTCGCCGCAGAAGT. The PCR products were treated with Cloning Enhancer (Clontech) and subjected to InFusion cloning reactions with the linearized *pattB-Gal4-hsp70* vector. We transformed the DNA constructs into Stellar competent cells (Clontech) and validated clones by Sanger sequencing^81^. Purified constructs were subjected to *PhiC31* transformation^83–86^ in the *Drosophila* strain of genotype *y w P*[*int*, *y* ^+^]; *P*[*attP2, y* ^+^] where the attP2 landing site is located at 68A4 on the 3^rd^ chromosome, by Model System Injections (Durham, NC). We identified positive transformants and, using balancer chromosomes and visible eye markers, created homozygous *Gal4-attP2-blw*-promoter flies. These were crossed to a homozygous *UAS*-luciferase reporter line. Protein lysates were extracted from sexes separately with 1X Luciferase Cell Culture Lysis Reagent (Promega) and quantified using the Bio-Rad DC Protein Assay kit II (Bio-Rad). The promoter activities were assessed with the Steady-Glo Luciferase Assay System (Promega) on a GloMax luminometer and data were analyzed using SAS software version 9.3^82^. We performed an ANOVA for luciferase activity, separately for males and females with form: *Y* = *μ* + *H* + *S* + *H* × *S* + *ε*, where *H* and *S* are haplotype and sex, respectively, and *ε* is the residual (error) variance. The normalized relative light units emitted by the assay revealed the strength of each promoter.

### Knockdown of *blw* using RNAi

A *UAS*-*blw*-RNAi line (ID = 34664) was obtained from the Vienna Drosophila Resource Center (VDRC)^87^. These flies were crossed to flies containing either an ubiquitin driver (*Gal4*-Ubi156) or an actin driver (*Gal4*-Actin) to disrupt *blw* expression. The progenitor VIE-260B genotype was also crossed to both drivers as a control.

### Overexpression of *blw* cDNA

To overexpress *blw* in flies, we amplified *blw* cDNA from the Drosophila reference strain 2057 and cloned it into the *pUAST-attb* vector at the *Not1/Xba1* restriction sites. The *pUAST-attB-blw*-cDNA purified construct was subjected to *PhiC31* transformation^83–86^ to the Drosophila strain having the following genotype: *y w P*[*int, y* ^+^]; *P*[*attP2, y* ^+^] where the attP2 landing site is located at 68A4 on the 3^rd^ chromosome (Model System Injections; Durham, NC). The injected G0 flies were crossed to a 2^nd^ and 3^rd^ chromosome balancer line (*w* ^*1118*^ *iso CSB; CyO/Sp*; *TM3, Sb/H*) and the G1 progeny were selected for the orange/red-eyed and Cy phenotype (positive transformants). *w* ^*1118*^ *iso CSB* is an isogenic *X* chromosome from the Canton S B (CSB) strain. Positive male transformants (G1) from G0 males were crossed to virgin females from a 3^rd^ chromosome balancer line (*w* ^*1118*^ *iso CSB; 2 iso CSB; TM3, Sb/H*) and the resulting F1 flies (*w* ^*1118*^ *iso CSB; 2 iso CSB/Cy*; *P*[*attP2*, *y* ^+^ *blw-cDNA w* ^+^]*/Sb*) were screened for red-eyed flies with the Cy and Sb phenotypes. *2 iso CSB* is an isogenic 2^nd^ chromosoems from the CSB strain Siblings were crossed to create homozygous flies of genotype *w* ^*1118*^ *iso CSB*; *2 iso CSB; P*[*attP2, y* ^+^ *blw-cDNA w* ^+^]). The homozygous flies were crossed to homozygous *Gal4* driver lines (*w* ^*1118*^ *iso CSB*; *2 iso CSB*; *P*[*attP2, y* ^+^ *Gal4-blw-GT*(*or AG*) *w* ^+^]) and the lifespans of the resulting progeny were measured. As control for lifespan, we used F1 progeny from the cross between *w* ^*1118*^ *iso CSB*; *2 iso CSB*; *P*[*attP2, y* ^+^] and the Ubiquitin driver line, *w* ^*1118*^ *iso CSB*; *2 iso CSB;Ubi-Gal4*[*156*].

### Lifespan measurements

Flies were generated for each *blw* promoter haplotype under controlled adult density conditions, by allowing 6 males and 6 females to mate and lay eggs for one day in vials containing 10 ml cornmeal-molasses-agar medium (cornmeal, 65 g/L; molasses, 45 ml/L; yeast, 13 g/L) under a 12 h light-dark cycle. Offspring from these vials were collected at 1–3 days post-eclosion for lifespan assays. Lifespan was assessed for each haplotype using 48 replicate vials, each containing 3 males and 3 females on 5 ml culture medium. We transferred flies without anesthesia every 2–3 days to new vials containing 5 ml of fresh food. We removed dead flies upon observation and recorded deaths every 1–3 days until all individuals were deceased.

To assess statistical significance for differences in lifespan, we fitted a Cox mixed effects model, where the hazard function is determined by fixed effects for sex, haplotype and the interaction between sex and haplotype, and random effects replicate within haplotype and sex by replicate effects. The model was fitted using the ‘coxme’ library in R^88^. We further performed a stratified analysis in each sex separately. Assumption of the hazard proportionality was checked using the ‘cox.zph’ function in the ‘survival’ package in R and was found to be met for the models fitted.

### Quantitative real time PCR

Total RNA was extracted from the progeny of the *Gal4*-*blw-*promoter *x UAS-blw* cDNA lines and the *Gal4*-ubiquitin x *y,w, P*[*int, y* ^+^]; *P*[*attP2, y* ^+^] control line. We synthesized cDNA from 120 ng of total RNA using the iScript cDNA synthesis kit (Bio-Rad) and performed quantitative RT-PCR using the Maxima SYBR Green/ROX qPCR master mix (Thermo Scientific) with the following primer pair specific to *blw* cDNA, 5′-ATGCAGACCGGTATCAAGG and 5′-GACGGTGGAACGCTTCTG. GAPDH was used as the internal control. The expression levels for *blw*-cDNA when driven by the *blw*-promoters were normalized against the control line to account for endogenous *blw* expression. The data were analyzed using the comparative C_T_ (threshold cycle) method^58^. We performed an ANOVA for *blw* expression levels of form: *Y* = *μ* + *H* + *S* + *H* × *S* + *ε*, where *H* and *S* are line and sex, respectively, and *ε* is the residual (error) variance. ANOVAs were performed using SAS software version 9.3^82^.

### Data availability statement

All relevant data are contained within the manuscript. Additional raw data will be available upon request.
